# Micro- and Nanostructured Biomaterials for Biomedical Applications and Regenerative Medicine

**DOI:** 10.3390/nano14221845

**Published:** 2024-11-18

**Authors:** Michele Bianchi, Gianluca Carnevale

**Affiliations:** 1 Department of Life Sciences, University of Modena and Reggio Emilia, 41125 Modena, Italy; 2 Department of Surgery, Medicine, Dentistry and Morphological Sciences with Interest in Transplant, Oncology and Regenerative Medicine, University of Modena and Reggio Emilia, 41124 Modena, Italy; gianluca.carnevale@unimore.it

## 1. Introduction

Over the past two decades, research on innovative micro- and nano-biomaterials has seen a significant surge in the bioengineering, biomedicine, and regenerative medicine fields. Multifunctional materials, featuring electrical conductivity, magnetic properties, micro- and nanostructured surfaces, smart 3D scaffolds, and smart implant coatings, represent just a portion of the vast and largely unexplored potential of nanomaterial-based applications [1,2,3,4]. Advanced biomaterials, with defined micro- and nanotopography, surface chemistry, and electrical, mechanical, and thermal properties, can be tailored to create nanoscale environments conducive to stem cell adhesion, proliferation, and differentiation [5,6,7,8,9]. These controlled environments promote optimal implant or device integration and guide regenerative processes [10,11,12]. In combination with the choice of advanced biomaterials, it is prominent to use the most appropriate cell sources, including stem cells, since they allow for the investigation of the physiological processes occurring during tissue regeneration [13,14,15,16,17]. In this regard, it is noteworthy that stem cells exert immunomodulatory abilities, and these properties might be affected by both the biomaterials and the surrounding microenvironment [18,19].

The aim of this Special Issue entitled “Micro- and Nanostructured Biomaterials for Biomedical Applications and Regenerative Medicine” was to gather and publish articles that highlight the impact of multifunctional properties of biomaterials at the micro- and nanoscale, with the goal of better characterizing the efficiency and functionality of novel biomaterials. Thirteen articles have been published, including two review articles and one brief report. The articles cover a broad range of topics, encompassing the design of novel materials for dental implants (n° 1 and 11), bone tissue regeneration (n° 3 and 6), functionalized nanoparticles for regenerative medicine (n° 2, 5, 12 and 13), antitumoral (n° 8 and 10), antibacterial (n° 7), and sensor (n° 4 and 9) applications.

## 2. An Overview of Published Articles

(1) Tagliaferri et al. [20] showed that CERID implants, made of a titanium core with a microstructured zirconium dioxide ceramic layer, offer better corrosion resistance, biocompatibility, and bone deposition over traditional titanium implants. In particular, CERID implants maintained surface integrity after cleaning, promoted osteogenic potential, and reduced inflammation compared to titanium, indicating CERID as a promising material for dental implants.

(2) Al-Saran et al. [21] investigated Lepidium sativum (LS) seed-extract-loaded solid lipid nanoparticles (SLNps) to enhance bioavailability and protect SH-SY5Y neuronal cells from oxidative stress and amyloid-toxicity associated with Alzheimer’s disease. These SLNps were formulated with LS extract, hyaluronic acid, and chia seed fatty acids. Characterization results indicated that LS-SLNps significantly improved cell proliferation and mitochondrial function while reducing oxidative stress, promoting neuroprotection, and reversing neurodegenerative effects by upregulating Wnt 3a/5a pathways and nerve growth factors.

(3) Gomez-Vazquez et al. [22] studied the physicochemical properties of three hydroxyapatite (HAp) types for bone tissue engineering: bovine-derived HAp-HB, chemically synthesized HAp-SC, and bioinspired HAp-SE from eggshells, all calcined under identical conditions at various temperatures. TGA revealed distinct thermal transitions based on source and composition. Elemental analysis showed similar calcium, phosphorus, and magnesium content among the samples. Microscopy techniques such as SEM and TEM indicated that crystallite size increased with temperature, and XRD demonstrated phase changes and crystallite growth. FTIR and Raman spectroscopy identified functional groups and dehydroxylation effects, with bioinspired HAp-SE showing partial phase changes and phosphorus interactions at elevated temperatures.

(4) Jelen et al. [23] successfully conjugated SARS-CoV-2 S1 spike protein fragments with gold nanoparticles (AuNPs) synthesized via Ultrasonic Spray Pyrolysis (USP), producing highly pure, round-shaped AuNPs with modifiable surfaces. The conjugation mechanism was investigated, and gel electrophoresis confirmed binding indirectly. XPS identified peptide bonds between the AuNPs and S1 protein fragments via a citrate stabilizer. The conjugate exhibited the expected response in a prototype lateral flow immunoassay, indicating the potential of USP-synthesized AuNPs for colorimetric and electrochemical sensors and immunoassay tests.

(5) Parikh et al. [24] explored bio-inspired all-carbon structures featuring carbon nanotube (CNT) carpets covalently attached to flexible carbon fabric for applications in wound healing and regenerative medicine. Covalent binding reduces cytotoxicity risks linked to loose CNTs, and the hierarchical architecture mimics natural materials, enhancing durability and biocompatibility. Tests confirmed the scaffold’s cytotoxicity, cell proliferation, and migration capabilities. Additionally, these scaffolds provided UVB protection, with cell growth tunable by adjusting CNT carpet height and surface wettability, showcasing promise for wound healing applications.

(6) In D’Amora et al.’s article [25], gellan gum (GG) was chemically modified with methacrylic groups to create a photocrosslinkable biomaterial ink named methacrylated GG (GGMA) with enhanced properties for 3D printing. GGMA was functionalized with either eumelanin or HAp nanoparticles. Various GGMA formulations were processed by 3D printing to produce scaffolds with organized structures. In vitro studies indicated that eumalanin promoted osteoblast proliferation and HAp enhanced alkaline phosphatase activity, suggesting the potential of these inks for bone tissue engineering scaffolds.

(7) Delgado et al. [26] examined how reaction time, temperature, and surface stabilizer concentration influence the thermal and bactericidal properties of cotton fabric coated with silver nanoparticles (AgNPs). The quality of the coating was assessed and correlated with synthesis parameters through experiments measuring heat transfer and bactericidal efficacy against various bacterial strains. The findings established relationships between AgNP size and shape, their agglomerates’ behavior, and the thermal barrier properties provided by surface modifiers like PVP.

(8) Busa et al. [27] focused on developing multifunctional drug delivery systems to combat drug-resistant cancer cells. Mesoporous silica nanoparticles (MSNs) loaded with curcumin were synthesized using a simple one-pot co-condensation method. These nanoparticles were modified to immobilize the prodrug Cisplatin on their carboxylate-modified surface, enabling both photodynamic therapy and chemotherapy for multidrug-resistant cancer cells. The hybrid nanocomposites demonstrated excellent structural properties, effectively internalized via endocytosis, and successfully delivered curcumin and cisplatin into the cytosol. Notably, curcumin within the MSNs enhanced cellular reactive oxygen species levels upon light irradiation, resulting in strong anti-cancer effects against drug-resistant cancer cells through the synergistic combination of chemo- and photodynamic therapies.

(9) Feng et al. [28] developed a smart self-regulatory insulin (INS) release system using erythrocytes (ERs) as carriers, designed to release INS in response to fluctuations in blood glucose levels, aiming to enhance patient compliance and health. To address ER availability and infection risks, a closed-loop autologous ER-mediated delivery (CAER) platform was created using a modified commercial hemodialysis instrument integrated with a glucose-responsive ER-based INS delivery system. In vivo tests demonstrated that the CAER platform responsively controlled INS release, effectively managing hyperglycemia and maintaining glucose levels within the normal range for up to three days without the added burden of hemodialysis in diabetic nephropathy rabbits.

(10) González-Pedroza et al. [29] explored the biosynthesis of AgNPs using extracts from Annona muricata, known for its anti-cancer properties. Antiproliferative effects were evaluated on breast, colon, and melanoma cancer cell lines, revealing that the fruit peel extract exhibited a more potent antitumor effect than the leaf extract, with effective cancer cell destruction achieved at concentrations not exceeding 3 g/mL.

(11) Li et al. [30] reviewed the applications of zeolites and zeolitic imidazolate frameworks (ZIFs) in dentistry. Common zeolite compounds used include silver, zinc, calcium, and strontium, while ZIFs like ZIF-8 and ZIF-67 have been utilized across various dental applications. These materials serve as antimicrobial additives in restorative materials, are incorporated into root-end fillings and denture bases, and act as antibacterial agents for periodontal pockets.

(12) Bekkouche et al. [31] provided a review of the applications of DNA-containing nanomaterials across multiple fields, including medicine and environmental science. Their unique properties, such as small size and strong permeability, enhance their value for diverse applications. Despite advancements, challenges remain in achieving precise cluster control and targeted drug delivery.

(13) Pisani et al. [32] reported on the targeted delivery of nanoparticles by modifying their surface with tailored chemical moieties. By functionalizing biocompatible polymer nanoparticles with CCL5, an inflammatory chemokine, they demonstrated selective internalization by CCR5+ monocytes in peripheral blood mononuclear cells, suggesting a promising approach for efficient delivery to specific leukocyte populations.

## 3. Conclusions

This Special Issue of *Nanomaterials* explores a diverse array of biomaterials designed for various biomedical applications, including nanostructured coatings, multifunctional nanoparticles, biomimetic devices, and hydrogel-based drug carriers, which are poised to serve as foundational elements for the next generation of biomaterials. While it does not encompass the vast range of available microstructured and nanostructured biomaterials and solutions for biomedical use, this Special Issue offers a concise overview of some of the most promising advancements in this fast-evolving, interdisciplinary field. Collectively, these studies drive innovation in biomaterials, drug delivery systems, and healthcare applications, highlighting progress in enhancing effectiveness, safety, and specificity.

## Data Availability

Not applicable.
